# Supplementary material for: DMRforPairs: identifying Differentially Methylated Regions between unique samples using array based methylation profiles
Source: BMC Bioinformatics. 2014 May 15;15:141. doi: 10.1186/1471-2105-15-141 (PMC4046028; doi:10.1186/1471-2105-15-141)
Supplement: Additional file 2 — DMRforPairs output for the comparison of A431-MCF7 and NA17018-NA17105. Please start from the HTML files in each folder. Available via the BMC Bioinformatics website. [file 1471-2105-15-141-S2.zip › 1394847754114233_MOESM2_ESM/A431_MCF7/figures/39469.pdf]

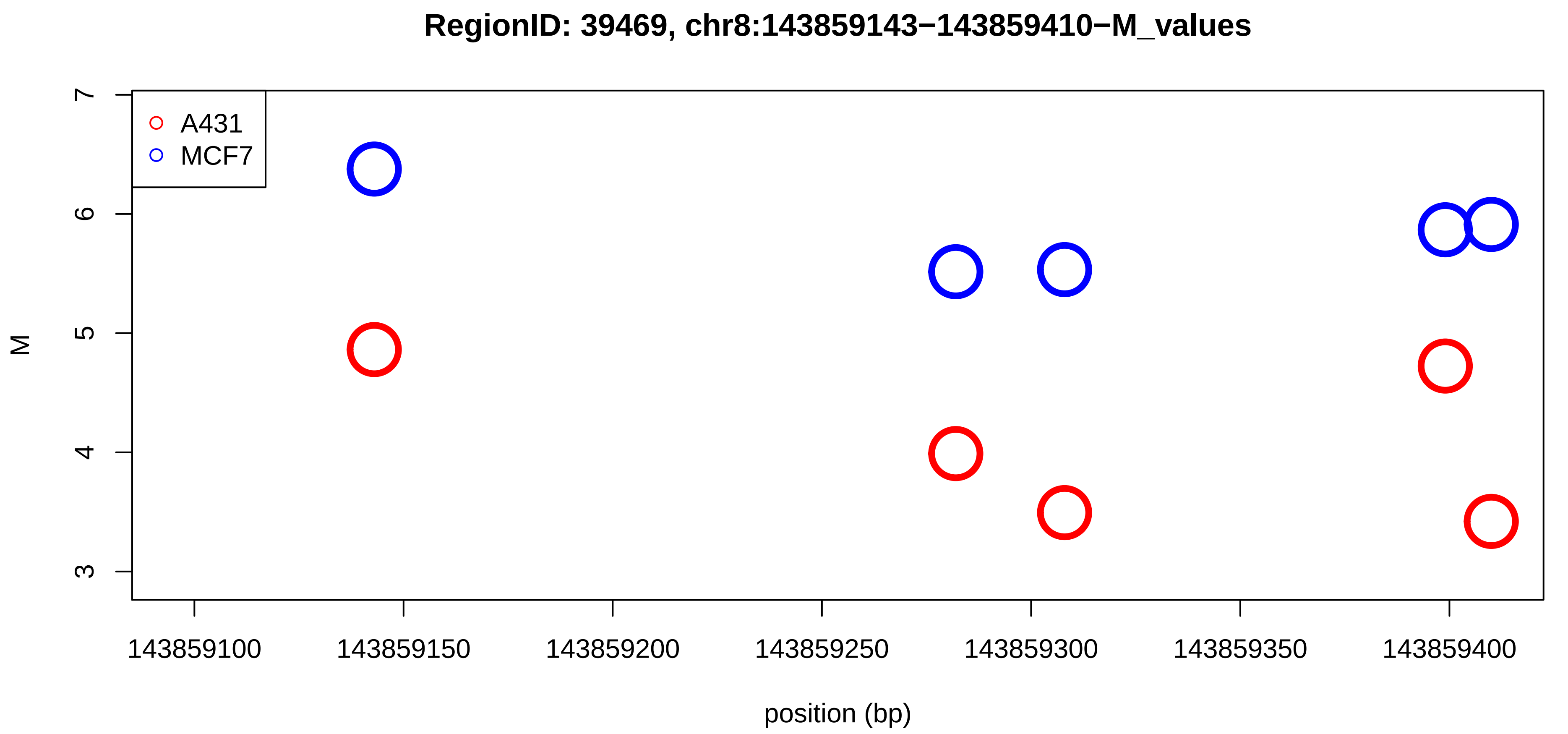
RegionID: 39469, chr8:143859143−143859410−M_values
A431
MCF7
M
3
4
5
6
7
143859100
143859150
143859200
143859250
143859300
143859350
143859400
position (bp)

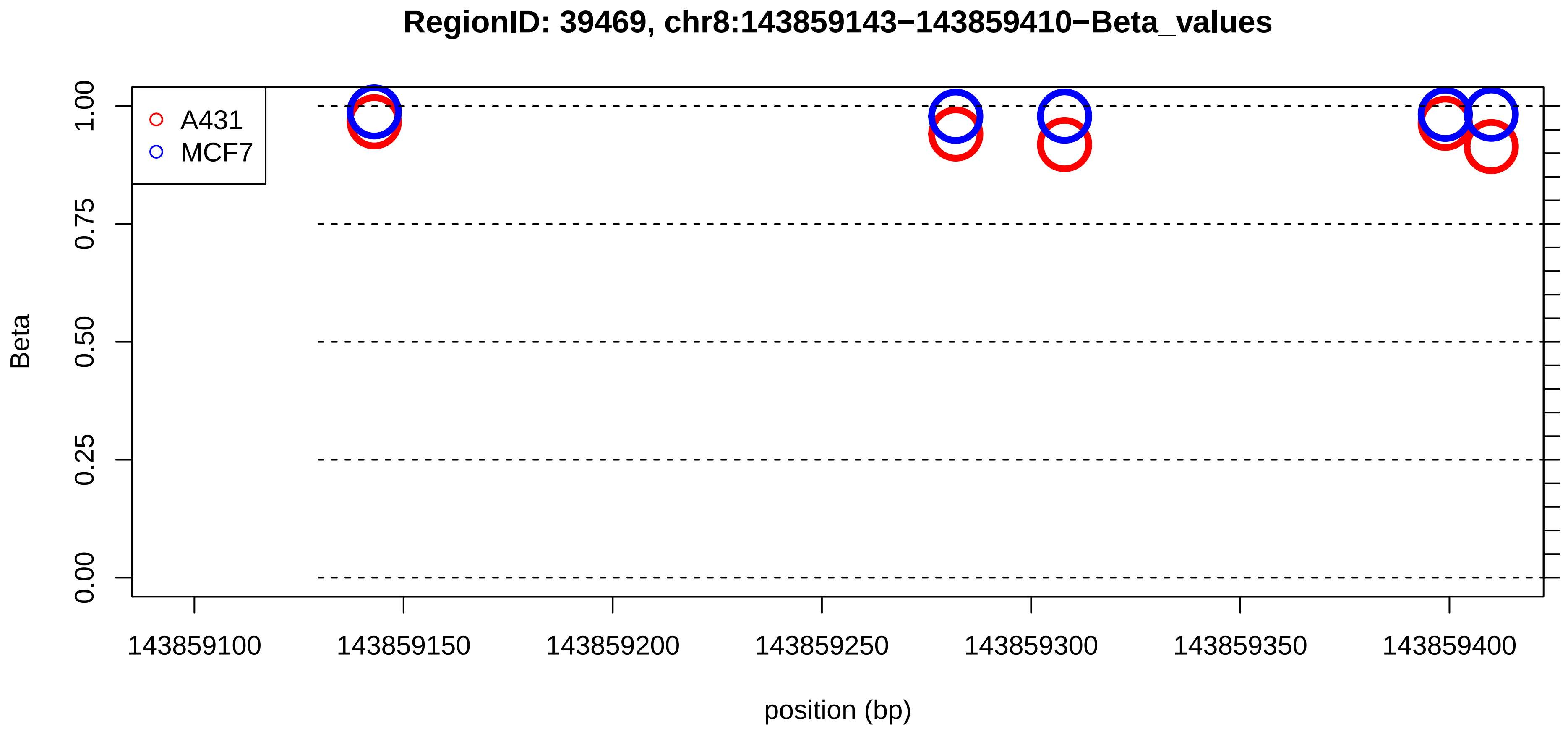
RegionID: 39469, chr8:143859143−143859410−Beta_values
A431
MCF7
Beta
0.00
0.25
0.50
0.75
1.00
143859100
143859150
143859200
143859250
143859300
143859350
143859400
position (bp)

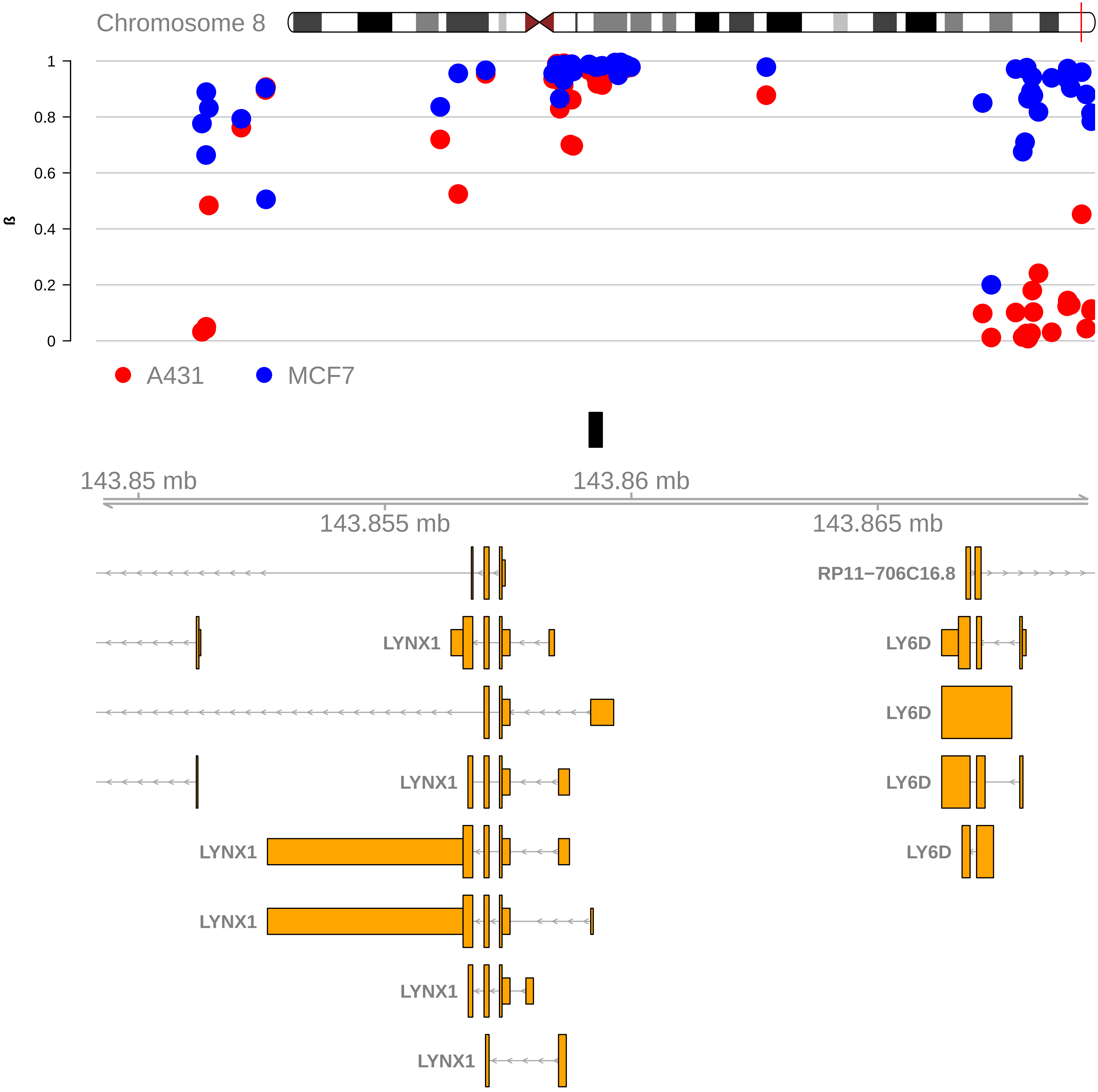

Chromosome 8
ß
1
0.8
0.6
0.4
0.2
0
A431
MCF7
143.85 mb
143.86 mb
143.855 mb
143.865 mb
RP11−706C16.8
LYNX1
LY6D
LY6D
LYNX1
LY6D
LYNX1
LY6D
LYNX1
LYNX1
LYNX1
